# Supplementary material for: The evolution of phenotypic plasticity when environments fluctuate in time and space
Source: Evol Lett. 2019 Jan 31;3(1):15–27. doi: 10.1002/evl3.100 (PMC6369965; doi:10.1002/evl3.100)
Supplement: Supplementary file 1 — Figure A‐1: Evolutionary time‐series of mean plasticity (left) and mean intercept (right) in the islands with the most extreme environments of development (left) and selection (right) over 15,000 generations Figure A‐2: Mean plasticity (b¯) in stochastic simulations with 1,000 islands over 10,000 generations. Figure A‐3: Mean plasticity (b¯) in stochastic simulations with 1,000 islands over 10,000 generations. Figure A‐4: Mean plasticity (b¯) in stochastic simulations with 1,000 islands over 10,000 generations. Figure A‐5: Mean plasticity (b¯) in stochastic simulations with 1,000 islands over 10,000 generations. [file EVL3-3-15-s001.pdf]

Supporting Information: The Evolution of Phenotypic Plasticity when Environments Fluctuate in Time and Space

Here we describe the model and how we derive the equilibrium conditions in more detail. To recap, we consider a trait whose phenotypic expression is determined by a linear reaction norm, with an intercept a that is invariant to the environment and a slope b that determines the degree to which the phenotype changes as the environment changes. The environment determining the plastic response is called the environment of development D and is different from the environment of selection S which determines the optimal value of the trait. The optimal trait value is also determined by a linear function with an intercept A and a slope B that determines the degree to which the optimum changes as the environment of selection changes.

Both environmental variables vary across the infinite number of islands in which the organisms live and also fluctuate in time according to an autoregressive process. Variation in the environment of development D and selection S is not independent but the degree of dependence can be different in time than it is in space. The reaction norm parameters evolve allowing the organisms to cope with these environmental fluctuations.

Generations are discrete with the order of events being fertilisation, development, selection, and then migration (of gametes). Phenotypes are assessed after development and for individual j on island i at time t is

$$z_{jit} = a_{jit} + a_{e_{jit}} + (b_{jit} + b_{e_{jit}})D_{it}, \tag{A-1}$$

where a_{jit} and $a_{e_{jit}}$ are the genetic and non-genetic components of the reaction norm intercept, and b_{jit} and $b_{e_{jit}}$ are the genetic and non-genetic components of the reaction norm slope. The within population variance of these terms is assumed constant in time and space and denoted as G^{aa} and G^{bb} for the genetic terms, and E^{aa} and E^{bb} for the environmental terms. In what follows we assume that any covariances between intercept and slope are zero (i.e. $G^{ab} = E^{ab} = 0$). Previous theoretical work generally assumes there is no environmental component to the reaction norm slope, but we include it here because decomposing between-individual variation in reaction norm slope into genetic and non-genetic components is an integral part of empirical studies (Nussey *et al.*, 2007). In the context of theoretical work its main role is to inflate the phenotypic variance in slope and phenotype which will be penalised under stabilising selection.

The optimal phenotype on island i at time t is given by

$$\theta_{z_{it}} = A + BS_{it}, \tag{A-2}$$

where intercept A represents the optimal phenotype in the reference (average) environment, and slope B the environmental sensitivity of the optimal phenotype (Chevin *et al.*, 2010).

Both environment variables can be decomposed into separable space-time processes, which for the environment of development is

$$D_{it} = D + D_i + D_t + D_{i \cdot t}, \tag{A-3}$$

where D denotes the grand mean, D_i the deviation of island i from the grand mean (averaged over time), D_t the deviation at time t from the grand mean (averaged over islands) and $D_{i \cdot t}$ the deviation specific to a time and place. Time is measured in units of generations.

Spatial components of the environmental variables D_i and S_i are assumed independent and identically distributed with corresponding variances $\sigma^2_{D_I}$ and $\sigma^2_{S_I}$, as are the space-time interaction components with variances $\sigma^2_{D_{I \cdot T}}$ and $\sigma^2_{S_{I \cdot T}}$. Temporal components are assumed to fluctuate according to an autoregressive process, giving rise to the recursive equation (for the environment of development)

$$D_{t+1} = \alpha_{D_T} D_t + \delta_{D_{t+1}}, \tag{A-4}$$

where α_{D_T} denotes the autocorrelation parameter, $\delta_{D_{t+1}}$ is a normally distributed increment with mean 0 and variance $(1 - \alpha^2_{D_T})\sigma^2_{D_T}$, and $\sigma^2_{D_T}$ is the stationary variance.

The environments of selection and development are assumed to be linearly associated in both space and time, such that the environment of selection can be expressed as a function of the environment of development:

$$S_i = \kappa_I \, D_i + e_{S_i} \tag{A-5}$$

$$S_t = \kappa_T \, D_t + e_{S_t}, \tag{A-6}$$

$$S_{i \cdot t} = \kappa_{I \cdot T} \, D_{i \cdot t} + e_{S_{i \cdot t}}, \tag{A-7}$$

where κ_I is the regression of S_i on D_i, κ_T the regression of S_t on D_t and $\kappa_{I \cdot T}$ the regression of $S_{i \cdot t}$ on $D_{i \cdot t}$. The products $B\kappa_I$ and $B\kappa_T$ are the DO-regressions in time and space and can differ if $\kappa_I \neq \kappa_T$. Residual errors of the spatial regression (e_{S_i}) have 0 mean and variance $\sigma^2_{e_{S_I}}$, and those of the space-time interaction regression ($e_{S_{i \cdot t}}$) have mean 0 and variance $\sigma^2_{e_{S_{I \cdot T}}}$. For the residuals of the temporal regression, we assume that both environmental variables have the same degree of temporal autocorrelation ($\alpha_{D_T} = \alpha_{S_T} = \alpha_T$), which implies

$$e_{S_{t+1}} = \alpha_T e_{S_t} + \delta_{e_{S_{t+1}}}, \tag{A-8}$$

where increments $\delta_{e_{S_{t+1}}}$ have mean 0 and variance $(1 - \alpha_T^2)\sigma^2_{e_{S_T}}$. $\sigma^2_{e_{S_T}}$ is the stationary residual variance and is equal to $\sigma^2_{S_T} - \kappa_T^2 \sigma^2_{D_T}$. We explore the assumption that $\alpha_{D_T} = \alpha_{S_T} = \alpha_T$ later.

The fitness of individual j on island i at time t with phenotype z_{jit} and plastic slope $b_{jit} + b_{e_{jit}}$ is described by the bivariate fitness function

$$W(z_{jit}, b_{jit} + b_{e_{jit}}) \propto \exp\Big[-\frac{(z_{jit} - \theta_{z_{it}})^2}{2\omega_z^2} - \frac{(b_{jit} + b_{e_{jit}} - \theta_{b_{it}})^2}{2\omega_b^2}\Big], \tag{A-9}$$

where $\theta_{z_{it}}$ is the optimal trait value, as described above, and $\theta_{b_{it}}$ is the optimal value of b in the population. We assume $\theta_{b_{it}} = 0 \; \forall \; \{i, t\}$, such that the absolute magnitude of plasticity is costly (van Tienderen, 1997; Lande, 2014; Kuijper & Hoyle, 2015). ω_z and ω_b are the widths of the bivariate fitness function along the phenotype and plasticity axis, respectively.

With a bivariate Gaussian fitness function, the selection gradient acting on the phenotype has a simple form (Lande, 1976):

$$\beta_{z_{it}} = \gamma_{z_{it}}(\theta_{z_{it}} - \bar{z}_{it}), \tag{A-10}$$

where $\theta_{z_{it}} - \bar{z}_{it}$ represents the deviation of the mean phenotype from the optimum, and $\gamma_{z_{it}} = \frac{1}{\omega_z^2 + P^{zz}_{it}}$ the strength of stabilising selection acting on the phenotype. P^{zz}_{it} is the phenotypic variance on island i at time t, given by

$$P^{zz}_{it} = G^{aa} + E^{aa} + 2(G^{ab} + E^{ab})D_{it} + (G^{bb} + E^{bb})D^2_{it}. \tag{A-11}$$

The selection gradient for plasticity is given by

$$\beta_{b_{it}} = -\gamma_b \bar{b}_{it}, \tag{A-12}$$

where $\bar{b}_{it}$ is the mean slope in the population and $\gamma_b = \frac{1}{\omega_b^2 + P^{bb}}$ is the strength of stabilising selection penalising slopes that deviate from 0. $P^{bb} = G^{bb} + E^{bb}$.

Traditionally, the expected evolutionary change in the reaction norm components (a and b) is derived by obtaining the selection gradients for these same reaction norm components Lande (e.g. 2009); Tufto (e.g. 2015). Here we take the unorthodox approach of including the redundant phenotype in the selection analysis and considering a trivariate model (phenotype, intercept, and slope). This allows us to put a cost on plasticity and so partition evolutionary change in plasticity into a direct response and an indirect response to selection on phenotype. Likewise, conditional on the phenotype z there is no direct selection on the intercepts a and so they evolve as a correlated response to selection on the phenotype (and the slope in situations where $G^{ab} \neq$). We denote the vector of average reaction norm components as $\bar{\mathbf{r}}_{it} = [\bar{z}_{it}, \bar{a}_{it}, \bar{b}_{it}]^\top$ and the selection gradient vector as $\boldsymbol{\beta}_{it} = [\beta_{z_{it}}, 0, \beta_{b_{it}}]^\top$. $\tilde{\mathbf{r}}_{it}$ denotes the vector of mean reaction norm components after selection but before migration, which is given by Lande (1979):

$$\tilde{\mathbf{r}}_{it} = \mathbf{G}_{it}\boldsymbol{\beta}_{it} + \bar{\mathbf{r}}_{it}, \tag{A-13}$$

where $\mathbf{G}_{it}$ is a matrix of genetic (co)variances:

$$\mathbf{G}_{it} = \begin{bmatrix} G^{zz}_{it} & G^{az}_{it} & G^{bz}_{it} \\ G^{az}_{it} & G^{aa} & G^{ab} \\ G^{bz}_{it} & G^{ab} & G^{bb} \end{bmatrix}. \tag{A-14}$$

We like the redundant approach because it allows us to put a cost on plasticity and so partition evolutionary change in plasticity into a direct response and an indirect response to selection on phenotype.

Although G^{aa}, G^{ab} and G^{bb} are assumed constant in time and space, (co)variances with z vary in time and space due to their dependency on D_{it}:

$$G_{it}^{zz} = G^{aa} + 2G^{ab}\,D_{it} + G^{bb}\,D_{it}^2 \tag{A-15}$$

$$G_{it}^{az} = G^{aa} + G^{ab}\,D_{it} \tag{A-16}$$

$$G_{it}^{bz} = G^{ab} + G^{bb}\,D_{it}. \tag{A-17}$$

Mean reaction norm components after both selection and migration are given by

$$\bar{\mathbf{r}}_{it+1} = \alpha_I \tilde{\mathbf{r}}_{it} + (1-\alpha_I)\frac{1}{n}\sum_{x=1}^{n}\tilde{\mathbf{r}}_{xt}, \tag{A-18}$$

where n is the number of islands and α_I the probability of philopatry $(1-m)$. Since migration does not alter the global expectation, then $\frac{1}{n}\sum\limits_{x=1}^{n}\tilde{\mathbf{r}}_{xt} = E_{i|t}[\tilde{\mathbf{r}}_{xt}] = E_{i|t}[\bar{\mathbf{r}}_{xt+1}]$, where $E_{i|t}[]$ is the expectation over islands at a particular time. This gives

$$\bar{\mathbf{r}}_{it+1} = \alpha_I(\mathbf{G}_{it}\boldsymbol{\beta}_{it} + \bar{\mathbf{r}}_{it}) + (1-\alpha_I)(\bar{\mathbf{r}} + \bar{\mathbf{r}}_{t+1}). \tag{A-19}$$

where $E_{i|t}[\bar{\mathbf{r}}_{xt+1}] = \bar{\mathbf{r}} + \bar{\mathbf{r}}_{t+1}$ following the notation of Equation A-3.

Equilibrium Conditions

We are interested in obtaining equilibrium distributions for the mean intercept and slope, and as a consequence the mean phenotypes. However, it is not possible to get analytical solutions for the model without making some additional assumptions and approximations. Throughout, we assume that intercepts and slopes are uncorrelated ($E^{ab} = G^{ab} = 0$) and that $\gamma_{z_{it}}$ is constant in time and space (and therefore denoted as γ_z). This latter approximation will hold if there is weak selection (because then P_{it}^{zz} is dominated by the constant ω_z^2) and/or if variation in the slopes is small (because then P_{it}^{zz} is dominated by the constants G^{aa} and E^{aa}). We also assume that variation in the mean slope over time within an island is small, which will be true if G^{bb} is small and/or temporal fluctuations are weak and not strongly autocorrelated. We relax these assumptions in a simulation model to assess the robustness of our conclusions.

At equilibrium, the expectation of $\bar{a}_{it}$ within each island should be constant over time, such that $E_{t|i}[\bar{a}_{it}] = E_{t|i}[\bar{a}_{it+1}]\ \forall\ \{i,t\}$ where $E_{t|i}[]$ is the expectation over time on a particular island (i.e. the conditional expectation of the variable conditional on island properties, that is, the variables D_i and S_i). When this condition is met (See Equations A-35 to A-40),

$$\bar{a} + \bar{a}_i = g_I(A + BS_i - \bar{a} - (\bar{b} + \bar{b}_i)D_i) + \bar{a}, \tag{A-20}$$

where $g_I = \frac{\alpha_I G^{aa}\gamma_z}{\alpha_I G^{aa}\gamma_z + (1-\alpha_I)}$. g_I takes values between 0 and 1, where 0 indicates no capacity to genetically track spatial fluctuations in the optimum and 1 indicates complete capacity to genetically track the optimum.

Likewise, at equilibrium, the expectation of $\bar{b}_{it}$ within each island should be constant over time, such that $E_{t|i}[\bar{b}_{it}] = E_{t|i}[\bar{b}_{it+1}]\,\forall\,\{i,t\}$, which gives (See Equations A-41 to A-43),

$$\bar{b} + \bar{b}_i = \frac{\alpha_I G^{bb}\gamma_z[D_i(A + BS_i - (\bar{a} + \bar{a}_i)) + B(\kappa_T\sigma_{D_T}^2 + \kappa_{I\cdot T}\sigma_{D_{I\cdot T}}^2) - Cov_{t|i}(\bar{a}_{it}, D_{it})] + (1-\alpha_I)\bar{b}}{\alpha_I G^{bb}[\gamma_z(\sigma_{D_T}^2 + D_i^2 + \sigma_{D_{I\cdot T}}^2) + \gamma_b] + 1 - \alpha_I}. \tag{A-21}$$

Equation A-21 includes a term for the covariance between the mean intercept and the environment of development over time within island i ($Cov_{t|i}(\bar{a}_{it}, D_{it})$). At equilibrium, this covariance is stationary, such that

$$Cov_{t|i}(\bar{a}_{it}, D_{it}) = Cov_{t|i}(\bar{a}_{it+1}, D_{it+1})\,\forall\,\{i,t\}, \tag{A-22}$$

which gives (See Equations A-44 to A-47)

$$Cov_{t|i}(\bar{a}_{it},\ D_{it}) = g_T\sigma_{D_T}^2[B\kappa_T - \bar{b} - \bar{b}_i], \tag{A-23}$$

where $g_T = \frac{\alpha_T G^{aa}\gamma_z}{\alpha_T G^{aa}\gamma_z + (1-\alpha_T)}$ and has the same form as g_I. This result relies on the two environmental variables having the same temporal autocorrelation ($\alpha_{D_T} = \alpha_{S_T} = \alpha_T$) which is discussed below. More generally, this result relies on the environmental variables experienced by an individual to be

independent of those experienced by more distant ancestors after conditioning on the environmental variables of the parents. Consequently, it is unclear to what degree these results would hold if the environment did not have this Markovian property (for example if the environment followed a moving average process rather than an autoregressive process).

To obtain solutions for this system of equations, we also need expressions for $\bar{a}$ and $\bar{b}$, which are the expectations of Equations A-20 and A-21 over islands. In both cases, we can take a Taylor expansion of Equations A-20 and A-21 around the mean environmental variables. For the mean intercept $\bar{a}$, a first order expansion is exact. For the mean slope $\bar{b}$, an exact expression is not obtainable and so we use a second order approximation as in Tufto (2000). *SpaceTime.nb* is a mathematica notebook for obtaining the solutions. In the main text we also give expressions for the temporal covariance between $\bar{a}_{it}$ and the environment of selection, and the spatial covariance between $\bar{a}_{it}$ and the environment of selection. The derivation of these covariances are given in Equations A-48 to A-51.

Our main result is an expression for mean plasticity under the limit $G^{bb} \to 0$. In this limit, there is sufficient genetic variance in plasticity for it to evolve to a non-trivial equilibrium, but once reached the strength of plasticity will fluctuate little in time or space and will be roughly constant:

$$\bar{b} = B\,\frac{\kappa_I(1-g_I)\sigma^2_{D_I} + \kappa_T(1-g_T)\sigma^2_{D_T} + \kappa_{I\cdot T}\sigma^2_{D_{I\cdot T}}}{\frac{\gamma_b}{\gamma_z} + (1-g_I)\sigma^2_{D_I} + (1-g_T)\sigma^2_{D_T} + \sigma^2_{D_{I\cdot T}}} \tag{A-24}$$

Although not pursued, the derivations seem to imply that if we allowed within-island temporal autocorrelation for $D_{i\cdot t}$ and $S_{i\cdot t}$ (rather than assuming white noise: $\alpha_{I\cdot T} = 0$), then $\sigma^2_{D_{I\cdot T}}$ would be multiplied by the comparable term $1 - g_{I\cdot T}$ in both the numerator and denominator.

In the main text we use Equation A-24 to obtain inequalities for when hyperplasticity and negative plasticity evolve. For hyperplasticity to evolve we get

$$\begin{aligned}
\bar{b}\frac{Cov(D_i,S_i)}{\sigma^2_{S_I}} &> B \\
B\,\frac{(1-g_I)Cov(S_i,D_i)+(1-g_T)Cov(S_t,D_t)+Cov(S_{i\cdot t},D_{i\cdot t})}{\frac{\gamma_b}{\gamma_z}+(1-g_I)\sigma^2_{D_I}+(1-g_T)\sigma^2_{D_T}+\sigma^2_{D_{I\cdot T}}} \\
\times\frac{Cov(D_i,S_i)}{\sigma^2_{S_I}} &> B \\
\frac{(1-g_I)Cov(S_i,D_i)+(1-g_T)Cov(S_t,D_t)+Cov(S_{i\cdot t},D_{i\cdot t})}{\frac{\gamma_b}{\gamma_z}+(1-g_I)\sigma^2_{D_I}+(1-g_T)\sigma^2_{D_T}+\sigma^2_{D_{I\cdot T}}} \\
\times\frac{Cov(D_i,S_i)}{\sigma^2_{S_I}} &> 1 \\
[(1-g_I)Cov(S_i,D_i) + (1-g_T)Cov(S_t,D_t) \\
+Cov(S_{i\cdot t},D_{i\cdot t})]\frac{Cov(D_i,S_i)}{\sigma^2_{S_I}} &> \frac{\gamma_b}{\gamma_z} + (1-g_I)\sigma^2_{D_I} \\
&\quad +(1-g_T)\sigma^2_{D_T} + \sigma^2_{D_{I\cdot T}} \\
(1-g_I)Cov(S_i,D_i)\frac{Cov(D_i,S_i)}{\sigma^2_{S_I}} - (1-g_I)\sigma^2_{D_I} &> \frac{\gamma_b}{\gamma_z} + (1-g_T)\sigma^2_{D_T} \\
&\quad -(1-g_T)Cov(S_t,D_t)\frac{Cov(D_i,S_i)}{\sigma^2_{S_I}} \\
&\quad +\sigma^2_{D_{I\cdot T}} - Cov(S_{i\cdot t},D_{i\cdot t})\frac{Cov(D_i,S_i)}{\sigma^2_{S_I}} \\
(1-g_I)\sigma^2_{D_I}(r_I^2-1) &> \frac{\gamma_b}{\gamma_z} + (1-g_T)\sigma^2_{D_T} \\
&\quad -(1-g_T)Cov(S_t,D_t)\frac{Cov(D_i,S_i)}{\sigma^2_{S_I}} \\
&\quad +\sigma^2_{D_{I\cdot T}} - Cov(S_{i\cdot t},D_{i\cdot t})\frac{Cov(D_i,S_i)}{\sigma^2_{S_I}} \\
(1-g_I)\sigma^2_{D_I}(r_I^2-1) &> \frac{\gamma_b}{\gamma_z} + (1-g_T)\sigma^2_{D_T} \\
&\quad \times(1-\frac{Cov(S_t,D_t)Cov(D_i,S_i)}{\sigma^2_{S_I}\sigma^2_{D_T}}) \\
&\quad +\sigma^2_{D_{I\cdot T}}(1-\frac{Cov(S_{i\cdot t},D_{i\cdot t})Cov(D_i,S_i)}{\sigma^2_{S_I}\sigma^2_{D_{I\cdot T}}}) \\
(1-g_I)\sigma^2_{D_I}(r_I^2-1) &> \frac{\gamma_b}{\gamma_z} + (1-g_T)\sigma^2_{D_T} \\
&\quad \times(1-r_T r_I\frac{\sigma_{S_T}\sigma_{D_I}}{\sigma_{D_T}\sigma_{S_I}}) \\
&\quad +\sigma^2_{D_{I\cdot T}}(1-r_{I\cdot T}r_I\frac{\sigma_{S_{I\cdot T}}\sigma_{D_I}}{\sigma_{D_{I\cdot T}}\sigma_{S_I}}) \\
-(1-g_I)\sigma^2_{D_I}(1-r_I^2) &> \frac{\gamma_b}{\gamma_z} + (1-g_T)\sigma^2_{D_T} \\
&\quad \times(1-r_T r_I\frac{\sigma_{S_T}\sigma_{D_I}}{\sigma_{D_T}\sigma_{S_I}}) \\
&\quad +\sigma^2_{D_{I\cdot T}}(1-r_{I\cdot T}r_I\frac{\sigma_{S_{I\cdot T}}\sigma_{D_I}}{\sigma_{D_{I\cdot T}}\sigma_{S_I}})
\end{aligned} \tag{A-25}$$

The LHS has to be non-positive because r^2 and g must lie between 0 and 1. Since $r_T = \kappa_T\frac{\sigma_{D_T}}{\sigma_{S_T}}$,

$r_I = \kappa_I \frac{\sigma_{D_I}}{\sigma_{S_I}}$ and $r_{I \cdot T} = \kappa_{I \cdot T} \frac{\sigma_{D_{I \cdot T}}}{\sigma_{S_{I \cdot T}}}$

$$-(1-g_I)\sigma^2_{D_I}(1-\kappa^2_I\frac{\sigma^2_{D_I}}{\sigma^2_{S_I}}) > \quad \frac{\gamma_b}{\gamma_z} + (1-g_T)\sigma^2_{D_T}(1-\kappa_T\kappa_I\frac{\sigma^2_{D_I}}{\sigma^2_{S_I}}) + \sigma^2_{D_{I\cdot T}}(1-\kappa_{I\cdot T}\kappa_I\frac{\sigma^2_{D_I}}{\sigma^2_{S_I}}). \quad \text{(A-26)}$$

For negative plasticity to evolve we get

$$\begin{aligned} \bar{b}\frac{Cov(D_i,S_i)}{\sigma^2_{S_I}} &< 0 \\ B\,\frac{\kappa_I(1-g_I)\sigma^2_{D_I}+\kappa_T(1-g_T)\sigma^2_{D_T}+\kappa_{I\cdot T}\sigma^2_{D_{I\cdot T}}}{\frac{\gamma_b}{\gamma_z}+(1-g_I)\sigma^2_{D_I}+(1-g_T)\sigma^2_{D_T}+\sigma^2_{D_{I\cdot T}}}\frac{Cov(D_i,S_i)}{\sigma^2_{S_I}} &< 0 \\ \frac{\kappa_I(1-g_I)\sigma^2_{D_I}+\kappa_T(1-g_T)\sigma^2_{D_T}+\kappa_{I\cdot T}\sigma^2_{D_{I\cdot T}}}{\frac{\gamma_b}{\gamma_z}+(1-g_I)\sigma^2_{D_I}+(1-g_T)\sigma^2_{D_T}+\sigma^2_{D_{I\cdot T}}}\frac{Cov(D_i,S_i)}{\sigma^2_{S_I}} &< 0 \end{aligned} \quad \text{(A-27)}$$

The denominator $\frac{\gamma_b}{\gamma_z} + (1-g_I)\sigma^2_{D_I} + (1-g_T)\sigma^2_{D_T} + \sigma^2_{D_{I\cdot T}}$ always has to be positive because g_T and g_I lie between 0 and 1. As such, inequality A-27 becomes

$$\begin{aligned} \frac{\kappa_I(1-g_I)\sigma^2_{D_I}+\kappa_T(1-g_T)\sigma^2_{D_T}+\kappa_{I\cdot T}\sigma^2_{D_{I\cdot T}}}{\frac{\gamma_b}{\gamma_z}+(1-g_I)\sigma^2_{D_I}+(1-g_T)\sigma^2_{D_T}+\sigma^2_{D_{I\cdot T}}}\frac{Cov(D_i,S_i)}{\sigma^2_{S_I}} &< 0 \\ [\kappa_I(1-g_I)\sigma^2_{D_I}+\kappa_T(1-g_T)\sigma^2_{D_T}+\kappa_{I\cdot T}\sigma^2_{D_{I\cdot T}}]\frac{\kappa_I\sigma^2_{D_I}}{\sigma^2_{S_I}} &< 0 \\ [\kappa_I(1-g_I)\sigma^2_{D_I}+\kappa_T(1-g_T)\sigma^2_{D_T}+\kappa_{I\cdot T}\sigma^2_{D_{I\cdot T}}]\kappa_I &< 0 \\ \kappa^2_I(1-g_I)\sigma^2_{D_I}+\kappa_T\kappa_I(1-g_T)\sigma^2_{D_T}+\kappa_{I\cdot T}\kappa_I\sigma^2_{D_{I\cdot T}} &< 0 \end{aligned} \quad \text{(A-28)}$$

to give

$$\kappa^2_I(1-g_I)\sigma^2_{D_I} < \quad -\kappa_T\kappa_I(1-g_T)\sigma^2_{D_T} - \kappa_{I\cdot T}\kappa_I\sigma^2_{D_{I\cdot T}} \quad \text{(A-29)}$$

Negative plasticity in space would then be favoured when the relationship between the environments of development and selection in space (κ_I) differ in sign from that in time (κ_T and $\kappa_{I\dot{T}}$) and the capacity to genetically track spatial variation (g_I) is large.

Assuming $\alpha_{D_T} = \alpha_{S_T}$

The derivation of $Cov_{t|i}(\bar{a}_{it}, D_{it})$ (Equation A-23) requires the assumption that $\bar{a}_{it+1}$ is independent of $\delta_{D_{t+1}}$ which will generally be true if both D_t and S_t are independent of $\delta_{D_{t+1}}$ (evolution has no foresight). $\delta_{D_{t+1}}$ and D_t are independent by construction but this is not generally the case for $\delta_{D_{t+1}}$ and S_t. Consider the vector autoregressive model (Lütkepohl, 2005):

$$\begin{bmatrix} D_{t+1} \\ S_{t+1} \end{bmatrix} = \begin{bmatrix} \alpha_{D_T} & 0 \\ 0 & \alpha_{S_T} \end{bmatrix} \begin{bmatrix} D_t \\ S_t \end{bmatrix} + \begin{bmatrix} \delta_{D_{t+1}} \\ \delta_{S_{t+1}} \end{bmatrix} \quad \text{(A-30)}$$

What we need to know is whether D_{t+1} and S_t are conditionally independent given D_t. Designating the diagonal matrix of autoregression coefficients as $\mathbf{\Psi}$ and the covariance matrix of the increments as $\mathbf{\Sigma}_\delta$, then the stationary covariance $\mathbf{\Sigma}$ is given by the solution to the Lyapunov Equation:

$$(\mathbf{I} - \mathbf{\Psi} \otimes \mathbf{\Psi})vec(\mathbf{\Sigma}) = \quad vec(\mathbf{\Sigma}_\delta) \quad \text{(A-31)}$$

which gives:

$$\begin{aligned} vec(\mathbf{\Sigma}) &= (\mathbf{I} - \mathbf{\Psi} \otimes \mathbf{\Psi})^{-1} vec(\mathbf{\Sigma}_\delta) \\ vec(\mathbf{\Sigma}) &= \begin{bmatrix} 1-\alpha^2_{D_T} & 0 & 0 & 0 \\ 0 & 1-\alpha_{D_T}\alpha_{S_T} & 0 & 0 \\ 0 & 0 & 1-\alpha_{D_T}\alpha_{S_T} & 0 \\ 0 & 0 & 0 & 1-\alpha^2_{S_T} \end{bmatrix}^{-1} \begin{bmatrix} \sigma^2_{\delta_{D_t}} \\ \sigma_{\delta_{D_t},\delta_{S_t}} \\ \sigma_{\delta_{D_t},\delta_{S_t}} \\ \sigma^2_{\delta_{S_t}} \end{bmatrix} \\ \mathbf{\Sigma} &= \begin{bmatrix} \frac{\sigma^2_{\delta_{D_t}}}{1-\alpha^2_{D_T}} & \frac{\sigma_{\delta_{D_t},\delta_{S_t}}}{1-\alpha_{S_T}\alpha_{D_T}} \\ \frac{\sigma_{\delta_{D_t},\delta_{S_t}}}{1-\alpha_{S_T}\alpha_{D_T}} & \frac{\sigma^2_{\delta_{S_t}}}{1-\alpha^2_{S_T}} \end{bmatrix} \end{aligned} \quad \text{(A-32)}$$

The covariance between observations at time t and time $t+1$ is equal to $\mathbf{\Psi\Sigma}$ (Lütkepohl, 2005) which gives:

$$cov\left(\begin{bmatrix} D_t \\ S_t \\ D_{t+1} \end{bmatrix}\right) = \begin{bmatrix} \frac{\sigma^2_{\delta_{D_t}}}{1-\alpha^2_{D_T}} & \frac{\sigma_{\delta_{D_t},\delta_{S_t}}}{1-\alpha_{S_T}\alpha_{D_T}} & \alpha_{D_T}\frac{\sigma^2_{\delta_{D_t}}}{1-\alpha^2_{D_T}} \\ \frac{\sigma_{\delta_{D_t},\delta_{S_t}}}{1-\alpha_{S_T}\alpha_{D_T}} & \frac{\sigma^2_{\delta_{S_t}}}{1-\alpha^2_{S_T}} & \alpha_{S_T}\frac{\sigma_{\delta_{D_t},\delta_{S_t}}}{1-\alpha_{S_T}\alpha_{D_T}} \\ \alpha_{D_T}\frac{\sigma^2_{\delta_{D_t}}}{1-\alpha^2_{D_T}} & \alpha_{S_T}\frac{\sigma_{\delta_{D_t},\delta_{S_t}}}{1-\alpha_{S_T}\alpha_{D_T}} & \frac{\sigma^2_{\delta_{D_t}}}{1-\alpha^2_{D_T}} \end{bmatrix} \tag{A-33}$$

from which the covariance between S_t and D_{t+1} after conditioning on D_t (i.e. the covariance between $\delta_{D_{t+1}}$ and S_t) can be obtained using results for conditional normal distributions:

$$\alpha_{S_T}\frac{\sigma_{\delta_{D_t},\delta_{S_t}}}{1-\alpha_{S_T}\alpha_{D_T}} - \frac{\sigma_{\delta_{D_t},\delta_{S_t}}}{1-\alpha_{S_T}\alpha_{D_T}}\left(\frac{\sigma^2_{\delta_{D_t}}}{1-\alpha^2_{D_T}}\right)^{-1}\alpha_{D_T}\frac{\sigma^2_{\delta_{D_t}}}{1-\alpha^2_{D_T}} = (\alpha_{S_T}-\alpha_{D_T})\frac{\sigma_{\delta_{D_t},\delta_{S_t}}}{1-\alpha_{S_T}\alpha_{D_T}} \tag{A-34}$$

This term is only zero when $\alpha_{S_T} = \alpha_{D_T} = \alpha_T$. If $\alpha_{S_T} > \alpha_{D_T}$ then the covariance between S_t and $\delta_{D_{t+1}}$ will take the same sign as κ_T, and the opposite sign if $\alpha_{D_T} > \alpha_{S_T}$. Consequently, the temporal association between intercept and the environment of development (Equation A-23) will be greater in magnitude when $\alpha_{S_T} > \alpha_{D_T}$ and smaller in magnitude when $\alpha_{D_T} > \alpha_{S_T}$. Given the role that the temporal association between intercept and the environment of development plays in the evolution of plasticity (Equation A-21) situations where $\alpha_{S_T} > \alpha_{D_T}$ will tend to reduce the evolution of plasticity for dealing with temporal fluctuations.

Although we acknowledge that $\alpha_{S_T} = \alpha_{D_T}$ is an assumption that is unlikely to hold in nature, we would also like to stress that because we use a discrete spatial model (the island model) rather than a continuous space model, the homologous spatial terms (α_{D_I} and α_{S_I}) are constrained to be equal (α_I) because they just depend on migration. Consequently, by assuming $\alpha_{S_T} = \alpha_{D_T}$ we do not allow the temporal model to diverge from the spatial model simply because we model time as continuous (although generations are discrete) and space as discrete. Moving from an island model to a stepping-stone or 2-D spatial field model would be difficult, but because the two variables could have different spatial autocorrelation parameters then α_{D_I} and α_{S_I} could differ despite the constraint of a common dispersal parameter. We expect the arguments put forward to explain what would happen when $\alpha_S \neq \alpha_D$ in time would also hold in space.

Simulations

To test how accurate our approximations are, we simulated the process for 15,000 generations using a population of 1,000 islands. The basic scheme is to simulate environmental variables following equations A-3:A-8. Initially $\bar{a}_i$ and $\bar{b}_i$ are drawn from unit normals for each island, and their values in the following generation obtained using Equation A-19. β_{it} and $\mathbf{G}_{it}$ vary in time and space and are calculated following equations A-10:A-12 and equations A-14:A-17 respectively. The procedure is then repeated each generation. The first 5,000 generations were discarded to allow the process to reach equilibrium. The simulation was written in R and the code is available in the file *SpaceTime.Rmd* and viewable in html (*SpaceTime.html*).

In order to assess how robust our $G^{bb} \rightarrow 0$ approximation is for mean plasticity, we simulate the process with parameter values (unless otherwise stated) $\alpha_T = \alpha_I = 0.5$, $\sigma^2_{D_T} = \sigma^2_{S_T} = \sigma^2_{D_I} = \sigma^2_{S_I} = 1$, $\sigma^2_{D_I \cdot T} = \sigma^2_{S_I \cdot T} = 0$, $A = 0$, $B = 1$, $G^{aa} = E^{aa} = G^{bb} = E^{bb} = 1$, $\kappa_T = -0.8$, $\kappa_I = 0.8$ and $\omega_b = 3$. In Figure A-1 we plot four evolutionary trajectories as examples with an emphasis on trajectories that are likely to approach equilibrium slowly in order to justify a burn-in period of 5000 generations.

In the main text we ran simulations across 100 migration rates ranging from 0 to 1 in equally spaced intervals and for four different values of ω_z (1, 5, 10, 20). We reproduce this figure here also (Figure A-2). In order to place the values for ω_z in context we also calculated the average value of ω_z scaled by the within-population phenotypic variance (i.e. calculated $E[\omega_z/P^{zz}_{it}]$ from the simulations) which gives values 0.27, 1.36, 2.72 and 5.46. Johnson & Barton (2005) using empirical data from Kingsolver *et al.* (2001) suggest a median value for ω_z/P^{zz} of about 5 which is equivalent to our weak selection scenario. However, the median value is likely smaller (stronger selection) because of reporting problems (Stinchcombe *et al.*, 2008) although Johnson & Barton (2005) stress that sampling errors are likely to mean that the strength of stabilising selection is overestimated (see Morrissey & Hadfield, 2011, also) and that previous theoretical work often uses much higher vales for ω_z/P^{zz} (weaker selection).

In addition, we ran simulations across 100 values of κ_T ranging from -0.8 to 0.8 in equally spaced intervals for the four different values of ω_z (Figure A-3).

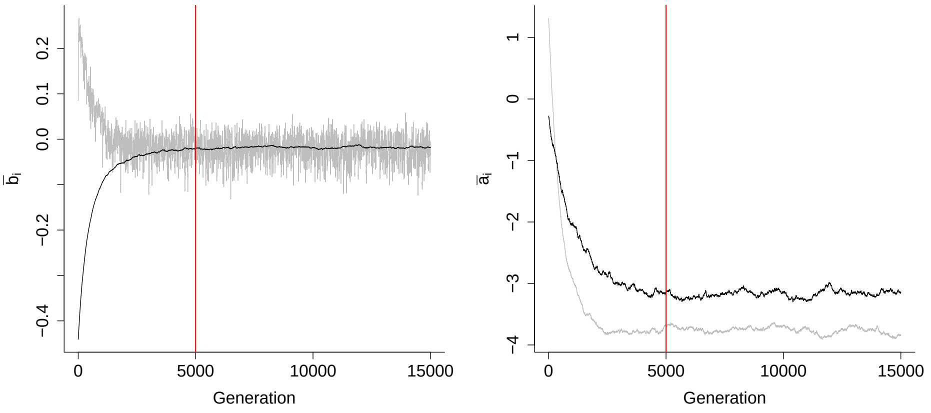

Figure A-1: Evolutionary time-series of mean plasticity (left) and mean intercept (right) in the islands with the most extreme environments of development (left) and selection (right) over 15,000 generations. Migration rate was set to zero $1 - \alpha_I = 0$ as this results in island-specific equilibria furthest away from the initial values and for which the time to equilibrium is likely to be highest. ω_z was set to 20 because weak selection is also likely to result in a slower approach to equilibrium. The black traces are for $G^{bb} = 0.01$; the smallest value of G^{bb} used in the simulations and for which the time to equilibrium should be longest. The grey traces are for when $G^{bb} = 1$. The red vertical bars indicate the end of the burn-in phase before which the samples are discarded.

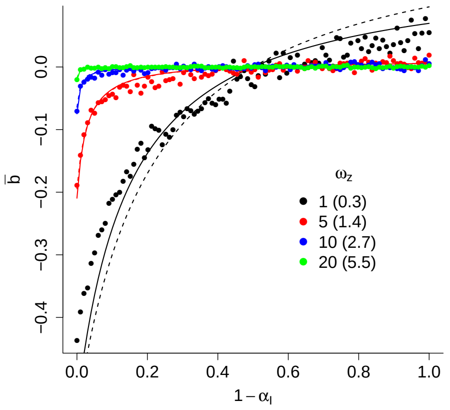

Figure A-2: Mean plasticity ($\bar{b}$) in stochastic simulations with 1,000 islands over 10,000 generations. A single simulation was conducted for each of 100 migration rates ($1-\alpha_I$) for four different strengths of stabilising selection on the phenotype (ω_z; small values indicate stronger stabilising selection). The number in parentheses is ω_z divided by the average within-population phenotypic variance. The expected mean plasticity obtained using the approximation $G^{bb} \to 0$ are shown where γ_z is set to $E[\gamma_{z_{it}}]$ calculated assuming no variance in slopes (dashed line) or a third-order Taylor expansion in D_{it} (solid line).

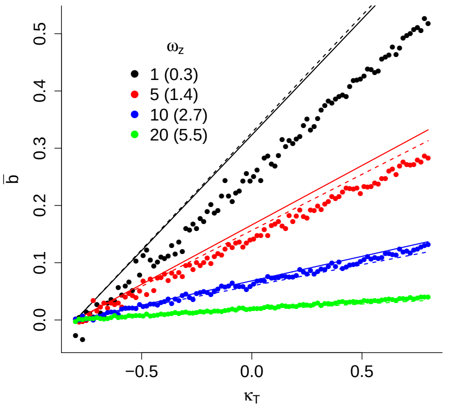

Figure A-3: Mean plasticity ($\bar{b}$) in stochastic simulations with 1,000 islands over 10,000 generations. A single simulation was conducted for each of 100 values of κ_T for four different strengths of stabilsing selection on the phenotype (ω_z; small values indicate stronger stabilising selection). The number in parentheses is ω_z divided by the average within-population phenotypic variance. The expected mean plasticity obtained using the approximation $G^{bb} \to 0$ are shown where γ_z is set to $E[\gamma_{z_{it}}]$ calculated assuming no variance in slopes (dashed line) or a third-order Taylor expansion in D_{it} (solid line).

The approximation is worse when $\kappa_T = \kappa_I$ so we held both at 0.8 and then ran simulations across 100 values of G^{bb} ranging from 0.01 to 1 in equally spaced intervals for the four different values of ω_z. E^{bb} was set to zero (Figure A-4).

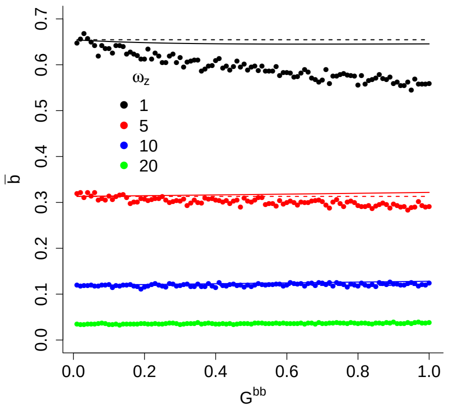

Figure A-4: Mean plasticity ($\bar{b}$) in stochastic simulations with 1,000 islands over 10,000 generations. A single simulation was conducted for each of 100 values of G^{bb} for four different strengths of stabilsing selection on the phenotype (ω_z; small values indicate stronger stabilising selection). $\kappa_T = \kappa_I = 0.8$ and $E^{bb} = 0$. The expected mean plasticity obtained using the approximation $G^{bb} \to 0$ are shown where γ_z is set to $E[\gamma_{z_{it}}]$ calculated assuming no variance in slopes (dashed line) or a third-order Taylor expansion in D_{it} (solid line).

Finally, we ran the same simulations as above but with P^{bb} ranging from 0.02 to 2 in equally spaced intervals for the four different values of ω_z. G^{bb} and E^{bb} were constrained to be the same (Figure A-5).

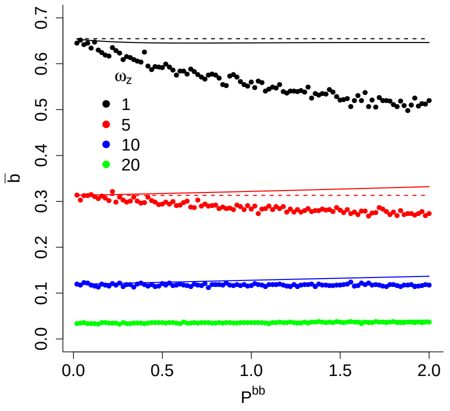

Figure A-5: Mean plasticity ($\bar{b}$) in stochastic simulations with 1,000 islands over 10,000 generations. A single simulation was conducted for each of 100 values of P^{bb} for four different strengths of stabilsing selection on the phenotype (ω_z; small values indicate stronger stabilising selection). $\kappa_T = \kappa_I = 0.8$ and G^{bb} and E^{bb} were constrained to be equal. The expected mean plasticity obtained using the approximation $G^{bb} \to 0$ are shown where γ_z is set to $E[\gamma_{z_{it}}]$ calculated assuming no variance in slopes (dashed line) or a third-order Taylor expansion in D_{it} (solid line).

Derivation of $\bar{a}_i$ (Equation A-20).

At equilibrium the following holds:

$$\begin{aligned}
E_{t|i}[\bar{a}_{it}] =& E_{t|i}[\bar{a}_{it+1}] \\
0 =& E_{t|i}[\bar{a}_{it+1}] - E_{t|i}[\bar{a}_{it}] \\
0 =& E_{t|i}[\bar{a}_{it+1} - \bar{a}_{it}]
\end{aligned} \tag{A-35}$$

From Equation A-19 this is equal to:

$$\begin{aligned}
0 =& E_{t|i}\left[\alpha_I[G^{az}_{it}\beta_{z_{it}} + \bar{a}_{it}] + (1-\alpha_I)(E_{i|t}[\bar{a}_{xt+1}]) - \bar{a}_{it}\right] \\
0 =& \alpha_I G^{aa} E_{t|i}[\beta_{z_{it}}] + \alpha_I E_{t|i}[\bar{a}_{it}] + (1-\alpha_I)E_{t|i}[E_{i|t}[\bar{a}_{xt+1}]] \\
& - E_{t|i}[\bar{a}_{it}] \\
0 =& \alpha_I G^{aa} E_{t|i}[\beta_{z_{it}}] + \alpha_I E_{t|i}[\bar{a}_{it}] + (1-\alpha_I)\bar{a} - E_{t|i}[\bar{a}_{it}] \\
(1-\alpha_I)E_{t|i}[\bar{a}_{it}] =& \alpha_I G^{aa} E_{t|i}[\beta_{z_{it}}] + (1-\alpha_I)\bar{a} \\
(1-\alpha_I)E_{t|i}[\bar{a}_{it}] =& \alpha_I G^{aa} E_{t|i}[\gamma_z(A + BS_{it} - \bar{a}_{it} - \bar{b}_{it}D_{it})] + (1-\alpha_I)\bar{a} \\
(1-\alpha_I)E_{t|i}[\bar{a}_{it}] =& \alpha_I G^{aa} E_{t|i}[\gamma_z(A + BS_{it} - \bar{b}_{it}D_{it})] - \alpha_I\gamma_z G^{aa} E_{t|i}[\bar{a}_{it}] \\
& + (1-\alpha_I)\bar{a} \\
E_{t|i}[\bar{a}_{it}](\alpha_I\gamma_z G^{aa} + (1-\alpha_I)) =& \alpha_I G^{aa} E_{t|i}[\gamma_z(A + BS_{it} - \bar{b}_{it}D_{it})] + (1-\alpha_I)\bar{a} \\
E_{t|i}[\bar{a}_{it}](\alpha_I\gamma_z G^{aa} + (1-\alpha_I)) =& \alpha_I\gamma_z G^{aa}(A + BE_{t|i}[S_{it}] - E_{t|i}[\bar{b}_{it}D_{it}]) + (1-\alpha_I)\bar{a} \\
E_{t|i}[\bar{a}_{it}](\alpha_I\gamma_z G^{aa} + (1-\alpha_I)) =& \alpha_I\gamma_z G^{aa}(A + BS_i - E_{t|i}[\bar{b}_{it}D_{it}]) + (1-\alpha_I)\bar{a} \\
E_{t|i}[\bar{a}_{it}] =& \frac{\alpha_I G^{aa}\gamma_z(A + BS_i - E_{t|i}[\bar{b}_{it}D_{it}]) + (1-\alpha_I)\bar{a}}{\alpha_I G^{aa}\gamma_z + (1-\alpha_I)}
\end{aligned} \tag{A-36}$$

Assuming G^{bb} is small such that $Cov_{t|i}[\bar{b}_{it}, D_{it}]$ is small then

$$\begin{aligned}
E_{t|i}[\bar{a}_{it}] =& \frac{\alpha_I G^{aa}\gamma_z(A + BS_i - E_{t|i}[\bar{b}_{it}]E_{t|i}[D_{it}]) + (1-\alpha_I)\bar{a}}{\alpha_I G^{aa}\gamma_z + (1-\alpha_I)} \\
E_{t|i}[\bar{a}_{it}] =& \frac{\alpha_I G^{aa}\gamma_z(A + BS_i - (\bar{b} + \bar{b}_i)D_i) + (1-\alpha_I)\bar{a}}{\alpha_I G^{aa}\gamma_z + (1-\alpha_I)}
\end{aligned} \tag{A-37}$$

Noting that

$$g_I = \frac{\alpha_I G^{aa}\gamma_z}{\alpha_I G^{aa}\gamma_z + (1-\alpha_I)} \tag{A-38}$$

and

$$\begin{aligned}
1 - g_I =& 1 - \frac{\alpha_I G^{aa}\gamma_z}{\alpha_I G^{aa}\gamma_z + (1-\alpha_I)} \\
1 - g_I =& \frac{\alpha_I G^{aa}\gamma_z + (1-\alpha_I)}{\alpha_I G^{aa}\gamma_z + (1-\alpha_I)} - \frac{\alpha_I G^{aa}\gamma_z}{\alpha_I G^{aa}\gamma_z + (1-\alpha_I)} \\
1 - g_I =& \frac{(1-\alpha_I)}{\alpha_I G^{aa}\gamma_z + (1-\alpha_I)}
\end{aligned} \tag{A-39}$$

gives Equation A-20:

$$\begin{aligned}
E_{t|i}[\bar{a}_{it}] =& g_I(A + BS_i - (\bar{b} + \bar{b}_i)D_i) + (1-g_I)\bar{a} \\
=& g_I(A + BS_i - \bar{a} - (\bar{b} + \bar{b}_i)D_i) + \bar{a}
\end{aligned} \tag{A-40}$$

Derivation of $\bar{b}_i$ (Equation A-21).

Following the same logic as above

$$\begin{aligned}
0 =&E_{t|i}[\alpha_I[G^{bz}_{it}\beta_{z_{it}} + G^{bb}\beta_{\bar{b}_{it}} + \bar{b}_{it}] \\
&+ (1-\alpha_I)(E_{i|t}[\bar{b}_{xt+1}]) - \bar{b}_{it}] \\
0 =&E_{t|i}\left[\alpha_I[G^{bb}D_{it}\beta_{z_{it}} + G^{bb}\beta_{\bar{b}_{it}} + \bar{b}_{it}]\right. \\
&+ (1-\alpha_I)(E_{i|t}[\bar{b}_{xt+1}]) - \bar{b}_{it} \\
0 =&\alpha_I G^{bb}E_{t|i}[D_{it}\beta_{z_{it}}] + \alpha_I G^{bb}E_{t|i}[\beta_{\bar{b}_{it}}] + \alpha_I E_{t|i}[\bar{b}_{it}] \\
&+ (1-\alpha_I)E_{t|i}[E_{i|t}[\bar{b}_{xt+1}]] - E_{t|i}[\bar{b}_{it}] \\
0 =&\alpha_I G^{bb}E_{t|i}[D_{it}\beta_{z_{it}}] + \alpha_I G^{bb}E_{t|i}[\beta_{\bar{b}_{it}}] + \alpha_I E_{t|i}[\bar{b}_{it}] \\
&+ (1-\alpha_I)\bar{b} - E_{t|i}[\bar{b}_{it}] \\
0 =&\alpha_I G^{bb}E_{t|i}[D_{it}\beta_{z_{it}}] - \alpha_I G^{bb}\gamma_b E_{t|i}[\bar{b}_{it}] + \alpha_I E_{t|i}[\bar{b}_{it}] \\
&+ (1-\alpha_I)\bar{b} - E_{t|i}[\bar{b}_{it}] \\
E_{t|i}[\bar{b}_{it}](1-\alpha_I+\alpha_I G^{bb}\gamma_b) =&\alpha_I G^{bb}E_{t|i}[D_{it}\beta_{z_{it}}] \\
&+ (1-\alpha_I)\bar{b} \\
E_{t|i}[\bar{b}_{it}](1-\alpha_I+\alpha_I G^{bb}\gamma_b) =&\alpha_I G^{bb}E_{t|i}[D_{it}\gamma_z(A+BS_{it}-\bar{a}_{it}-\bar{b}_{it}D_{it})] \\
&+ (1-\alpha_I)\bar{b} \\
E_{t|i}[\bar{b}_{it}](1-\alpha_I+\alpha_I G^{bb}\gamma_b) =&\alpha_I\gamma_z G^{bb}(AE_{t|i}[D_{it}] + BE_{t|i}[D_{it}S_{it}] - E_{t|i}[D_{it}\bar{a}_{it}] \\
&- E[\bar{b}_{it}D^2_{it}]) + (1-\alpha_I)\bar{b} \\
E_{t|i}[\bar{b}_{it}](1-\alpha_I+\alpha_I G^{bb}\gamma_b) =&\alpha_I\gamma_z G^{bb}(AE_{t|i}[D_{it}] \\
&+ BCov_{t|i}(D_{it},S_{it}) + BE_{t|i}[D_{it}]E_{t|i}[S_{it}] \\
&- Cov_{t|i}(D_{it},\bar{a}_{it}) - E_{t|i}[D_{it}]E_{t|i}[\bar{a}_{it}] \\
&- E_{t|i}[\bar{b}_{it}D^2_{it}]) + (1-\alpha_I)\bar{b} \\
E_{t|i}[\bar{b}_{it}](1-\alpha_I+\alpha_I G^{bb}\gamma_b) =&\alpha_I\gamma_z G^{bb}(AD_i + B(\kappa_T\sigma^2_{D_T} + \kappa_{I\cdot T}\sigma^2_{D_{I\cdot T}}) + BD_iS_i \\
&- Cov_{t|i}(D_{it},\bar{a}_{it}) - D_iE_{t|i}[\bar{a}_{it}] - E_{t|i}[\bar{b}_{it}D^2_{it}]) \\
&+ (1-\alpha_I)\bar{b} \\
E_{t|i}[\bar{b}_{it}](1-\alpha_I+\alpha_I G^{bb}\gamma_b) =&\alpha_I\gamma_z G^{bb}(D_i(A+BS_i-E_{t|i}[\bar{a}_{it}]) + B(\kappa_T\sigma^2_{D_T} + \kappa_{I\cdot T}\sigma^2_{D_{I\cdot T}}) \\
&- Cov_{t|i}(D_{it},\bar{a}_{it}) - E_{t|i}[\bar{b}_{it}D^2_{it}]) + (1-\alpha_I)\bar{b}
\end{aligned} \tag{A-41}$$

Again assuming $Cov_{t|i}(\bar{b}_{it}, D_{it})$ is small such that $E_{t|i}[\bar{b}_{it}D^2_{it}] \approx E_{t|i}[\bar{b}_{it}]E_{t|i}[D^2_{it}]$

$$\begin{aligned}
E_{t|i}[\bar{b}_{it}](1-\alpha_I+\alpha_I G^{bb}\gamma_b) =&\alpha_I\gamma_z G^{bb}(D_i(A+BS_i-E_{t|i}[\bar{a}_{it}]) \\
&+ B(\kappa_T\sigma^2_{D_T} + \kappa_{I\cdot T}\sigma^2_{D_{I\cdot T}}) \\
&- Cov_{t|i}(D_{it},\bar{a}_{it}) \\
&- E_{t|i}[\bar{b}_{it}]E_{t|i}[D^2_{it}])) \\
&+ (1-\alpha_I)\bar{b} \\
E_{t|i}[\bar{b}_{it}](1-\alpha_I+\alpha_I G^{bb}\gamma_b) =&\alpha_I\gamma_z G^{bb}(D_i(A+BS_i-E_{t|i}[\bar{a}_{it}]) \\
&+ B(\kappa_T\sigma^2_{D_T} + \kappa_{I\cdot T}\sigma^2_{D_{I\cdot T}}) \\
&- Cov_{t|i}(D_{it},\bar{a}_{it}) \\
&- E_{t|i}[\bar{b}_{it}](\sigma^2_{D_T} + \sigma^2_{D_{I\cdot T}} + D^2_i)) \\
&+ (1-\alpha_I)\bar{b} \\
E_{t|i}[\bar{b}_{it}](1-\alpha_I+\alpha_I G^{bb}(\gamma_b+\gamma_z(\sigma^2_{D_T} + \sigma^2_{D_{I\cdot T}} + D^2_i)) =&\alpha_I\gamma_z G^{bb}(D_i(A+BS_i-E_{t|i}[\bar{a}_{it}]) \\
&+ B(\kappa_T\sigma^2_{D_T} + \kappa_{I\cdot T}\sigma^2_{D_{I\cdot T}}) \\
&- Cov_{t|i}(D_{it},\bar{a}_{it})) + (1-\alpha_I)\bar{b}
\end{aligned} \tag{A-42}$$

to give

$$E_{t|i}[\bar{b}_{it}] = \frac{\alpha_I\gamma_z G^{bb}(D_i(A+BS_i-(\bar{a}+\bar{a}_i)) + B(\kappa_T\sigma^2_{D_T} + \kappa_{I\cdot T}\sigma^2_{D_{I\cdot T}}) - Cov_{t|i}(D_{it},\bar{a}_{it})) + (1-\alpha_I)\bar{b}}{(1-\alpha_I+\alpha_I G^{bb}(\gamma_b+\gamma_z(\sigma^2_{D_T} + \sigma^2_{D_{I\cdot T}} + D^2_i))} \tag{A-43}$$

Derivation of $Cov_{t|i}(\bar{a}_{it}, D_{it})$ (Equation A-23)

Since D and D_i are constants, and the genetic values at time $t+1$ are independent of δ_{Dt+1} (if $\alpha_{D_T} = \alpha_{S_T}$):

$$\begin{aligned} Cov_{t|i}(\bar{a}_{it},\ D_{it}) &= Cov_{t|i}(\bar{a}_{it+1},\ D_{it+1}) \\ &= Cov_{t|i}(\bar{a}_{it+1},\ D + D_i + \alpha_T D_t + \delta_{Dt+1}) \\ &= \alpha_T Cov_{t|i}(\bar{a}_{it+1}, D_t), \end{aligned} \tag{A-44}$$

Using this result,

$$\begin{aligned} Cov_{t|i}(\bar{a}_{it}, D_{it}) =& \alpha_T Cov_{t|i}\big(\alpha_I[G^{az}\beta_z + \bar{a}_{it}] + (1-\alpha_I)E_{i|t}[\bar{a}_{xt+1}], D_t\big) \\ =& \alpha_T\alpha_I Cov_{t|i}\big(G^{az}\beta_z + \bar{a}_{it}, D_t\big) + \alpha_T(1-\alpha_I)Cov_{t|i}(E_{i|t}[\bar{a}_{xt+1}], D_t) \\ =& \alpha_T\alpha_I Cov_{t|i}(G^{az}\beta_z, D_t) + \alpha_T\alpha_I Cov_{t|i}(\bar{a}_{it}, D_t) \\ &+ \alpha_T(1-\alpha_I)Cov_{t|i}(E_{i|t}[\bar{a}_{xt+1}], D_t) \\ =& \alpha_T\alpha_I G^{aa}\gamma_z Cov_{t|i}(A + BS_{it} - a_{it} - \bar{b}_{it}D_{it}, D_t) + \alpha_T\alpha_I Cov_{t|i}(\bar{a}_{it}, D_t\big) \\ &+ \alpha_T(1-\alpha_I)Cov_{t|i}(E_{i|t}[\bar{a}_{xt+1}], D_t) \\ =& \alpha_T\alpha_I G^{aa}\gamma_z Cov_{t|i}(A + BS_{it} - \bar{b}_{it}D_{it}, D_t) + \alpha_T\alpha_I(1-G^{aa}\gamma_z)Cov_{t|i}(\bar{a}_{it}, D_t\big) \\ &+ \alpha_T(1-\alpha_I)Cov_{t|i}(E_{i|t}[\bar{a}_{xt+1}], D_t) \\ =& \alpha_T\alpha_I G^{aa}\gamma_z Cov_{t|i}(A + BS_{it} - \bar{b}_{it}D_{it}, D_t) + \alpha_T\alpha_I(1-G^{aa}\gamma_z)Cov_{t|i}(\bar{a}_{it}, D_t\big) \\ &+ \alpha_T(1-\alpha_I)Cov_{t|i}(\bar{a}_{t+1}, D_t) \\ =& \alpha_T\alpha_I G^{aa}\gamma_z Cov_{t|i}(A + BS_{it} - \bar{b}_{it}D_{it}, D_t) + \alpha_T\alpha_I(1-G^{aa}\gamma_z)Cov_{t|i}(\bar{a}_{it}, D_t\big) \\ &+ \alpha_T(1-\alpha_I)Cov_{t|i}(\bar{a}_{t+1}, \frac{1}{\alpha_T}D_{t+1}) \\ =& \alpha_T\alpha_I G^{aa}\gamma_z Cov_{t|i}(A + BS_{it} - \bar{b}_{it}D_{it}, D_t) + \alpha_T\alpha_I(1-G^{aa}\gamma_z)Cov_{t|i}(\bar{a}_{it}, D_t\big) \\ &+ (1-\alpha_I)Cov_{t|i}(\bar{a}_{t+1}, D_{t+1}) \\ =& \alpha_T\alpha_I G^{aa}\gamma_z Cov_{t|i}(A + BS_{it} - \bar{b}_{it}D_{it}, D_t) + \alpha_T\alpha_I(1-G^{aa}\gamma_z)Cov_{t|i}(\bar{a}_{it}, D_t\big) \\ &+ (1-\alpha_I)Cov_{t|i}(\bar{a}_t, D_t) \end{aligned} \tag{A-45}$$

Under the previous assumption that $\bar{b}_{it}$ varies little within islands such that $Cov_{t|i}(\bar{b}_{it}D_{it}, D_t) = (\bar{b} + \bar{b}_i)\sigma^2_{D_T}$

$$\begin{aligned} Cov_{t|i}(\bar{a}_{it}, D_{it}) &= \alpha_T\alpha_I G^{aa}\gamma_z(B\kappa_T - (\bar{b} + \bar{b}_i))\sigma^2_{D_T} \\ &\quad + \alpha_T\alpha_I(1-G^{aa}\gamma_z)Cov_{t|i}(\bar{a}_{it}, D_t\big) \\ &\quad + (1-\alpha_I)Cov_{t|i}(\bar{a}_t, D_t) \\ Cov_{t|i}(\bar{a}_{it}, D_{it})(1 - \alpha_T\alpha_I(1-G^{aa}\gamma_z)) &= \alpha_T\alpha_I G^{aa}\gamma_z(B\kappa_T - (\bar{b} + \bar{b}_i))\sigma^2_{D_T} \\ &\quad + (1-\alpha_I)Cov_{t|i}(\bar{a}_t, D_t) \\ Cov_{t|i}(\bar{a}_{it}, D_{it})(1 - \alpha_T\alpha_I(1-G^{aa}\gamma_z) - (1-\alpha_I)) &= \alpha_T\alpha_I G^{aa}\gamma_z(B\kappa_T - (\bar{b} + \bar{b}_i))\sigma^2_{D_T} \\ Cov_{t|i}(\bar{a}_{it}, D_{it}) &= \frac{\alpha_T\alpha_I G^{aa}\gamma_z(B\kappa_T - (\bar{b} + \bar{b}_i))\sigma^2_{D_T}}{1 - \alpha_T\alpha_I(1-G^{aa}\gamma_z) - (1-\alpha_I)} \\ Cov_{t|i}(\bar{a}_{it}, D_{it}) &= \frac{\alpha_T G^{aa}\gamma_z(B\kappa_T - (\bar{b} + \bar{b}_i))\sigma^2_{D_T}}{1 - \alpha_T(1-G^{aa}\gamma_z)} \end{aligned} \tag{A-46}$$

to give Equation A-23:

$$Cov_{t|i}(\bar{a}_{it}, D_{it}) = g_T\alpha_T G^{aa}\gamma_z(B\kappa_T - (\bar{b} + \bar{b}_i))\sigma^2_{D_T} \tag{A-47}$$

Derivation of $Cov_{t|i}(\bar{a}_{it}, S_{it})$

Following the same logic as above

$$\begin{aligned}Cov_{t|i}(\bar{a}_{it}, S_{it}) &= \alpha_T\alpha_I G^{aa}\gamma_z Cov_{t|i}(A + BS_{it} - \bar{b}_{it}D_{it}, S_t) \\ &+ \alpha_T\alpha_I(1 - G^{aa}\gamma_z)Cov_{t|i}(\bar{a}_{it}, S_t) + (1-\alpha_I)Cov_{t|i}(\bar{a}_t, S_t) \\ &= \alpha_T\alpha_I G^{aa}\gamma_z(B\sigma^2_{S_t} - Cov_{t|i}(\bar{b}_{it}D_{it}, S_t)) \\ &+ \alpha_T\alpha_I(1 - G^{aa}\gamma_z)Cov_{t|i}(\bar{a}_{it}, S_t) + (1-\alpha_I)Cov_{t|i}(\bar{a}_t, S_t)\end{aligned} \tag{A-48}$$

and again assuming that $\bar{b}_{it}$ varies little within islands such that $Cov_{t|i}(\bar{b}_{it}D_{it}, S_t) \approx (\bar{b} + \bar{b}_i)\kappa_T\sigma^2_{D_t}$:

$$\begin{aligned}Cov_{t|i}(\bar{a}_{it}, S_{it}) &= \alpha_T\alpha_I G^{aa}\gamma_z(B\sigma^2_{S_t} - (\bar{b} + \bar{b}_i)\kappa_T\sigma^2_{D_t}) \\ &+ \alpha_T\alpha_I(1 - G^{aa}\gamma_z)Cov_{t|i}(\bar{a}_{it}, S_t) \\ &+ (1-\alpha_I)Cov_{t|i}(\bar{a}_t, S_t) \\ Cov_{t|i}(\bar{a}_{it}, S_{it})(1 - \alpha_T\alpha_I(1 - G^{aa}\gamma_z) - (1-\alpha_I)) &= \alpha_T\alpha_I G^{aa}\gamma_z(B\sigma^2_{S_t} - (\bar{b} + \bar{b}_i)\kappa_T\sigma^2_{D_t}) \\ Cov_{t|i}(\bar{a}_{it}, S_{it}) &= \frac{\alpha_T\alpha_I G^{aa}\gamma_z(B\sigma^2_{S_t} - (\bar{b} + \bar{b}_i)\kappa_T\sigma^2_{D_t})}{1 - \alpha_T\alpha_I(1 - G^{aa}\gamma_z) - (1-\alpha_I)} \\ Cov_{t|i}(\bar{a}_{it}, S_{it}) &= \frac{\alpha_T G^{aa}\gamma_z(B\sigma^2_{S_t} - (\bar{b} + \bar{b}_i)\kappa_T\sigma^2_{D_t})}{1 - \alpha_T(1 - G^{aa}\gamma_z)}\end{aligned} \tag{A-49}$$

to give

$$Cov_{t|i}(\bar{a}_{it}, S_{it}) = g_T(B\sigma^2_{S_t} - (\bar{b} + \bar{b}_i)\kappa_T\sigma^2_{D_t}) \tag{A-50}$$

Derivation of $Cov_{i|t}(\bar{a}_{it}, S_{it})$

Given $Cov_{i|t}(\bar{a}_{it}, S_{it}) = Cov_{i|t}(\bar{a}_i, S_i)$ and that the equilbrium solution for $\bar{a}_i$ is $g_I[BS_i - (\bar{b} + \bar{b}_i)D_i]$ we get

$$\begin{aligned}Cov_{i|t}(\bar{a}_{it}, S_{it}) &= Cov_{i|t}(g_I[BS_i - \bar{b}D_i], S_i) \\ &= g_I[B\sigma^2_{S_I} - \bar{b}\kappa_I\sigma^2_{D_I}]\end{aligned} \tag{A-51}$$

under the limit $G^{bb} \to 0$ where $\bar{b}_i = 0$.

References

Chevin, L.M., Lande, R. & Mace, G.M. (2010). Adaptation, plasticity, and extinction in a changing environment: Towards a predictive theory. *PLoS Biology*, 8, e1000357.

Johnson, T. & Barton, N. (2005). Theoretical models of selection and mutation on quantitative traits. *Philosophical Transactions of the Royal Society B: Biological Sciences Sciences*, 360, 1411–1425.

Kingsolver, J.G., Hoekstra, H.E., Hoekstra, J.M., Berrigan, D., Vignieri, S.N., Hill, C.E., Hoang, A., Gibert, P. & Beerli, P. (2001). The strength of phenotypic selection in natural populations. *American Naturalist*, 157, 245–261.

Kuijper, B. & Hoyle, R.B. (2015). When to rely on maternal effects and when on phenotypic plasticity? *Evolution*, 69, 950–968.

Lande, R. (1976). Natural selection and random genetic drift in phenotypic evolution. *Evolution*, 30, 314–334.

Lande, R. (1979). Quantitative genetic analysis of multivariate evolution, applied to the brain:body size allometry. *Evolution*, 33, 402–416.

Lande, R. (2009). Adaptation to an extraordinary environment by evolution of phenotypic plasticity and genetic assimilation. *Journal of Evolutionary Biology*, 22, 1435–1446.

Lande, R. (2014). Evolution of phenotypic plasticity and environmental tolerance of a labile quantitative character in a fluctuating environment. *Journal of evolutionary biology*, 27, 866–875.

Lütkepohl, H. (2005). *New introduction to multiple time series analysis.* Springer Science & Business Media.

Morrissey, M.B. & Hadfield, J.D. (2011). Directional selection in temporally replicated studies is remarkably consistent. *Evolution*, 66, 435–442.

Nussey, D.H., Wilson, A.J. & Brommer, J.E. (2007). The evolutionary ecology of individual phenotypic plasticity in wild populations. *Journal of Evolutionary Biology*, 20, 831–844.

Stinchcombe, J.R., Agrawal, A.F., Hohenlohe, P.A., Arnold, S.J. & Blows, M.W. (2008). Estimating nonlinear selection gradients using quadratic regression coefficients: double or nothing? *Evolution*, 62, 2435–2440.

van Tienderen, P.H. (1997). Generalists, specialists, and the evolution of phenotypic plasticity in sympatric populations of distinct species. *Evolution*, 51, 1372–1380.

Tufto, J. (2000). The evolution of plasticity and nonplastic spatial and temporal adaptations in the presence of imperfect environmental cues. *American Naturalist*, 156, 121–130.

Tufto, J. (2015). Genetic evolution, plasticity, and bet-hedging as adaptive responses to temporally autocorrelated fluctuating selection: A quantitative genetic model. *Evolution*, 69, 2034–2049.
